# Supplementary material for: The estimated burden of scrub typhus in Thailand from national surveillance data (2003-2018)
Source: PLoS Negl Trop Dis. 2020 Apr 14;14(4):e0008233. doi: 10.1371/journal.pntd.0008233 (PMC7182275; doi:10.1371/journal.pntd.0008233)
Supplement: S1 Document — GlobCover 2009 with a resolution of 1 km was used (http://due.esrin.esa.int/page_globcover.php). (DOCX) [file pntd.0008233.s001.docx]

**S2 Document**

Details of spatio-temporal analyses

*Spatial autocorrelation of scrub typhus cases*

Correlogram analysis and Moran's I test were performed to test the significance of spatial correlations using the packages spatialEco (Evans, 2015) and spData package (Bivand et al., 2018) for the whole-time range.

*Semi-variogram and kriging interpolation of scrub typhus cases*

Kriging involves using a fixed number of nearest neighbour points within a fixed radius (De Smith et al., 2015) and relies on semi-variograms that quantify spatial autocorrelation among all pairs of data according to distance (Isaaks & Srivastava, 1989). Semi-variograms were estimated by calculating the squared difference of scrub typhus cases between all pairs of sub-district centroid. Semi-variance values were grouped and averaged according to separation distance (lags). Semi-variogram model and kriging were computed using the packages FRK (Zammit-Mangion 2018) and INLA (Brownrigg et al., 2015).

*Temporal analysis and temporal auto-correlation*

We used time-series analysis to study the epidemiology of scrub typhus during the period 2003-2018. Exponential smoothing model was used to assess temporal trends in the overall rates of scrub typhus incidence using ncf implemented in R (Bjornstad ON, 2019). Residual ACF (Autocorrelation Function) was examined to determine the general form of the model to be fitted. Considering the ACF graphs, different ARIMA models were identified for model selection. The series were then decomposed with a moving average taking into account a period of one year (Kendall & Stuart, 1983).

Similarly, we analyzed the temporal variations of rainfall and temperature using the same methodology as above.

We used wavelet analysis which transforms function to decompose a time series to reveal periodic signals at each time point in the series. The wavelet analysis coefficients showed magnitudes of correlation of the scrub typhus cases for each year and period length and were displayed using a power spectrum over the full time series. We used the packages biwavelet (Gouhier et al., 2018) and WaveletComp (Roesch & Schmidbauer, 2015).

*Association between rainfall, temperature and scrub typhus cases*

We used the above time-series analysis to study the patterns of scrub typhus cases during the study period. Using ACF, we investigated correlation lag and the correlation values at the best lag period (in month) among rainfall, temperature and scrub typhus cases. The function ccf was used to compute the cross-correlation or cross-covariance between univariate series, i.e. rainfall and scrub typhus cases and temperature and scrub typhus cases.

*Spatial analysis of the land use*

We extracted information from the global land use cover for each sub-district using the packages raster (Hijmans, 2019), rgdal (Bivand et al., 2019), dismo (Hijmans et al., 2017) and rgeos (Bivand & Rundel, 2019). We extracted the size of each land use class and computed the following variables (see Supplementary Document S1 for the classification):

forest cover (%): as the sum of closed broadleaved deciduous forest, and closed needle-leaved evergreen forest, divided by the total area of the sub-district;

forest open cover (%): as the sum of closed to open broadleaved evergreen or semi-deciduous forest, and closed to open mixed broadleaved and needle-leaved forest, and open broadleaved deciduous forest, divided by the total area of the sub-district;

grassland open cover (%): as the sum of closed to open herbaceous vegetation, and closed to open grassland, divided by the total area of the sub-district;

mosaic habitat cover (%): as the sum of mosaic cropland, mosaic grassland, and mosaic vegetation, divided by the total area of the sub-district;

rain-fed cropland cover (%): as rain-fed croplands divided by the total area of the sub-district;

flooded-irrigated land cover (%): as post-flooding or irrigated croplands divided by the total area of the sub-district.

We estimated the complexity and the fragmentation of the land use within each sub-district with two metrics:

habitat complexity (shape.index): using the function PatchStat of the package SDMTools (van Der Wal et al., 2019), which measures the complexity of the land use and computed as the sum of each patches perimeter divided by the square root of patch area,

habitat fragmentation (frac.dim.index); using the function PatchStat, which reflects shape complexity across a range of spatial scales and computes as the logarithm of patch perimeter (m) divided by the logarithm of patch area.

*Association between scrub typhus cases and investigated factors*

The distribution of scrub typhus cases per sub-district and per month was investigated using the package fitdistrplus (Delignette-Muller & Dutang, 2015).

We ran a GLM model on scrub typhus cases and investigated the potential explanatory factors: land use characteristics (habitat complexity, habitat fragmentation), forest cover (%), forest open cover (%), grassland open cover (%), mosaic habitat cover (%), rain-fed land cover (%), flooded-irrigated land cover (%), population size, rainfall and temperature using a negative binomial link function and select the best model using the package MuMi (Bartoń, 2019) and Akaike Information Criteria (AIC) selection procedure. The uncertainty that the ‘‘best’’ model would emerge as superior if different data were used was quantified with Akaike weights wr (Burnham and Anderson, 2002). Potential collinearity were assessed using VIF (Variance Inflating Factor) using the package car (Fox & Weisberg, 2011). Odds ratios were computed using the package oddsratio (Schratz, 2017). Performance of models was assessed by estimating the percentage of deviance explained using maximum likelihood R^2^.

*Association between scrub typhus cases and investigated factors using General Additive Modeling*

General Additive Modeling (GAM) is an extension of the generalized linear models with the adaptability for non-normally distributed variables. The model assumes that the response variable, here scrub typhus cases per sub-district per month, is dependent on the univariate smooth-terms of independent variables (Hastie & Tibshirani, 1990). All models were fitted using the MGCV package (Wood, 2017). We used the function gam.check to choose the basis dimension for each predictor according to estimated degrees of freedom value in the main effect. Outputs of GAM models were obtained using the package gratia (Simpson, 2019).

References

Bartoń K (2019). MuMIn: Multi-Model Inference. R package version 1.43.6. <https://CRAN.R-project.org/package=MuMIn>

Bates D, Mächler M, Bolker B, Walker S. Fitting linear mixed-effects models using lme4. Journal of Statistical Software 2015; 67(1): 1-48.

Bivand R, Keitt T, Rowlingson B (2019). rgdal: Bindings for the 'Geospatial' Data Abstraction Library. R package version 1.4-4., https://CRAN.R-project.org/package=rgdal

Bivand R, Nowosad J, Lovelace R, Monmonier M, Snow G. (2018). Package 'spData'.

Bivand R and Rundel C (2019). rgeos: Interface to Geometry Engine - Open Source ('GEOS'). R package version 0.5-2. <https://cran.r-project.org/package=rgeos>

Bjornstad ON. (2019). ncf: Spatial Covariance Functions. R package version 1.2-8. <https://CRAN.R-project.org/package=ncf>

Brownrigg R, Minka T, Deckmyn A. maps: Draw Geographical Maps, R package version 3.0.1. 2015.

Burnham KP, Anderson DR (2002). Model Selection and Multimodel Inference: A Practical Information-Theoretic Approach. Second edition. New York; NY: Springer.

Delignette-Muller ML, & Dutang C. fitdistrplus: An R Package for Fitting Distributions. Journal of Statistical Software; 2015, 64(4): 1-34.

De Smith MJ, Goodchild MF, Longley P. Geospatial Analysis: A Comprehensive Guide to Principles, Techniques and Software Tools (5 ed.). 2015; Leicester, UK: Troubador Ltd.

Evans J (2015). spatialEco. R package version 2.0-0.

Fox J, Weisberg S (2011). An {R} Companion to Applied Regression, Second Edition. Thousand Oaks CA: Sage.

Gouhier TC, Grinsted A, Simko V. (2018). R package biwavelet: Conduct Univariate and Bivariate Wavelet Analyses (Version 0.20.17). <https://github.com/tgouhier/biwavelet>

Hastie TJ, Tibshirani RJ. Generalized Additive Models. 1990; New-York: Chapman and Hall – CRC.

Hijmans RJ (2019). raster: Geographic Data Analysis and Modeling. R package version 3.0-7. <https://CRAN.R-project.org/package=raster>

Hijmans RJ *et al*. (2017). dismo: Species Distribution Modeling. R package version 1.1-4. <https://cran.r-project.org/package=dismo>

Isaaks EH, Srivastava RM. An Introduction to Applied Geostatistics. 1989; Oxford: Oxford University Press.

Kendall, M., Stuart, A. and Ord, J.K. (1983). The Advanced Theory of Statistics. Vol. 3: Design and analysis and time-series. Griffin, London.

R Core Team. (2018). R: A Language and Environment for Statistical Computing. R Foundation for Statistical Computing, Vienna. https://www.R-project.org

Roesch A, Schmidbauer H. (2015). WaveletComp. http://www.hs-stat.com/projects/WaveletComp/WaveletComp_guided_tour.pdf

Schratz P. (2017). R package 'oddsratio': Odds ratio calculation for GAM(M)s & GLM(M)s, version: 1.0.2, doi: 10.5281/zenodo.1095472

Simpson GL. (2019). gratia: Graceful 'ggplot'-Based Graphics and Other Functions for GAMs Fitted Using 'mgcv'. R package version 0.2-8. <https://gavinsimpson.github.io/gratia>

Sing T, Sander O, Beerenwinkel N, Lengauer T (2005). ROCR: visualizing classifier performance in R. Bioinformatics 21(20): 7881.

Van der Wal J *et al*. (2019). SDMTools: Species Distribution Modelling Tools: Tools for processing data associated with species distribution modelling exercise. R package version 1.1-221.2. <https://cran.r-project.org/package=SDMTools>

Wood SN. (2017). Generalized Additive Models: An Introduction with R, 2^nd^ Edition, Chapman and Hall/CRC, New York. <https://doi.org/10.1201/9781315370279>

Zammit-Mangion A. (2018). FRK: Fixed Rank Kriging. R package version 0.2.2. [https://CRAN.R-project.org/package=FRK](https://cran.r-project.org/package=FRK)
