# Supplementary figures and images for: The estimated burden of scrub typhus in Thailand from national surveillance data (2003-2018)
Source: PLoS Negl Trop Dis. 2020 Apr 14;14(4):e0008233. doi: 10.1371/journal.pntd.0008233 (PMC7182275; doi:10.1371/journal.pntd.0008233)

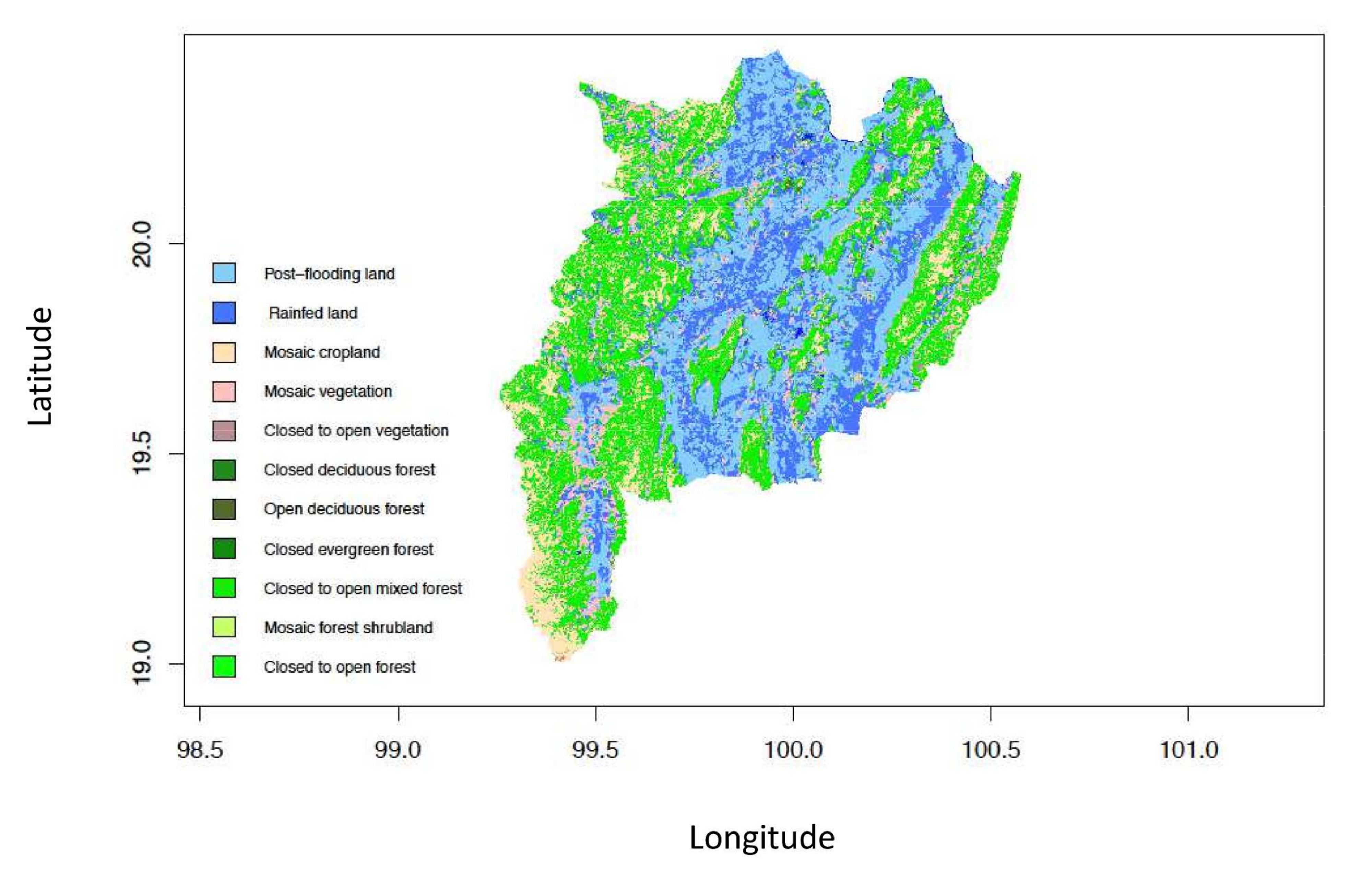

Supplement: S1 Fig — GlobCover 2009 satellite imagery was used. (TIF) [file pntd.0008233.s004.tif]

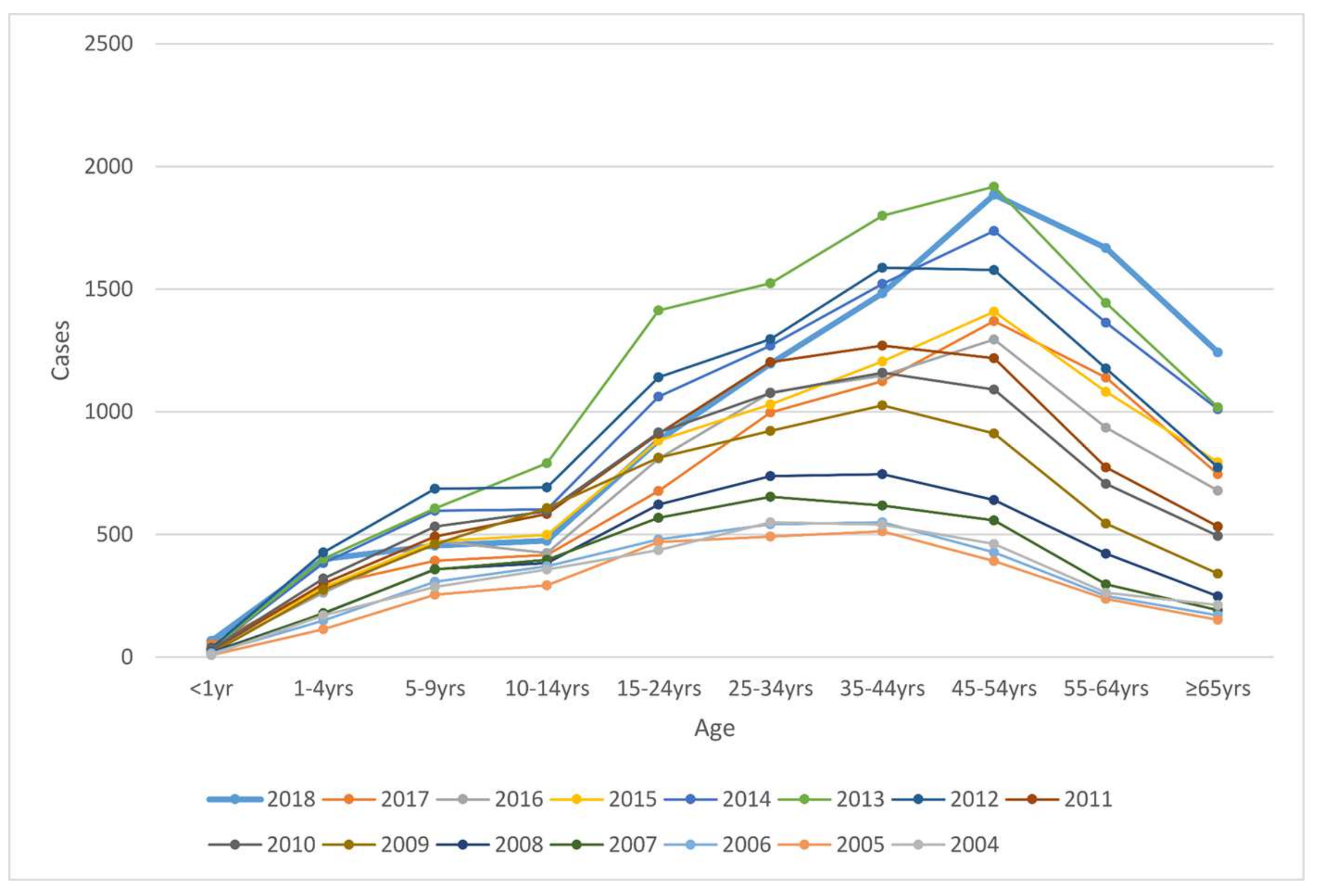

Supplement: S2 Fig — (TIF) [file pntd.0008233.s005.tif]

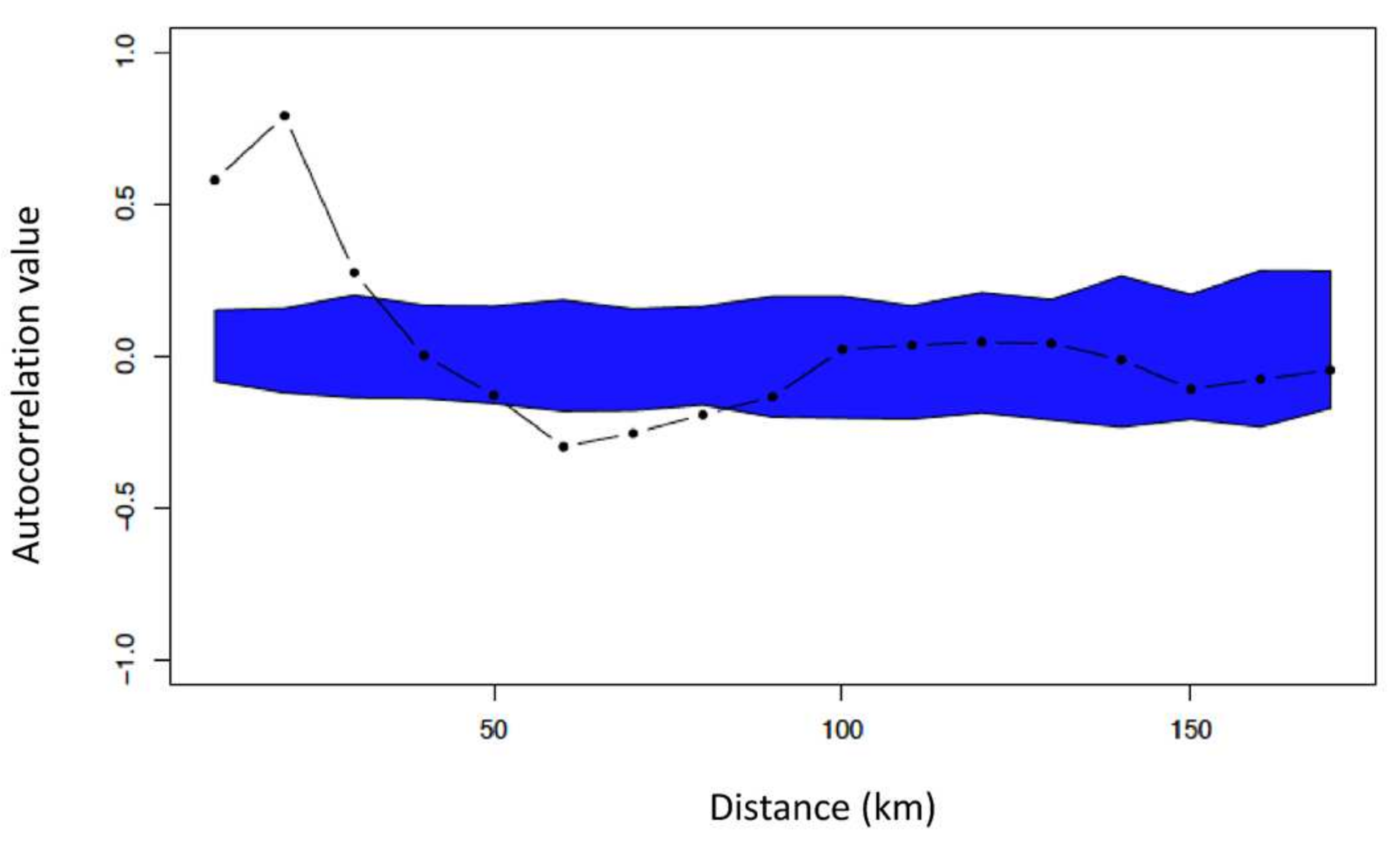

Supplement: S3 Fig — (TIF) [file pntd.0008233.s006.tif]

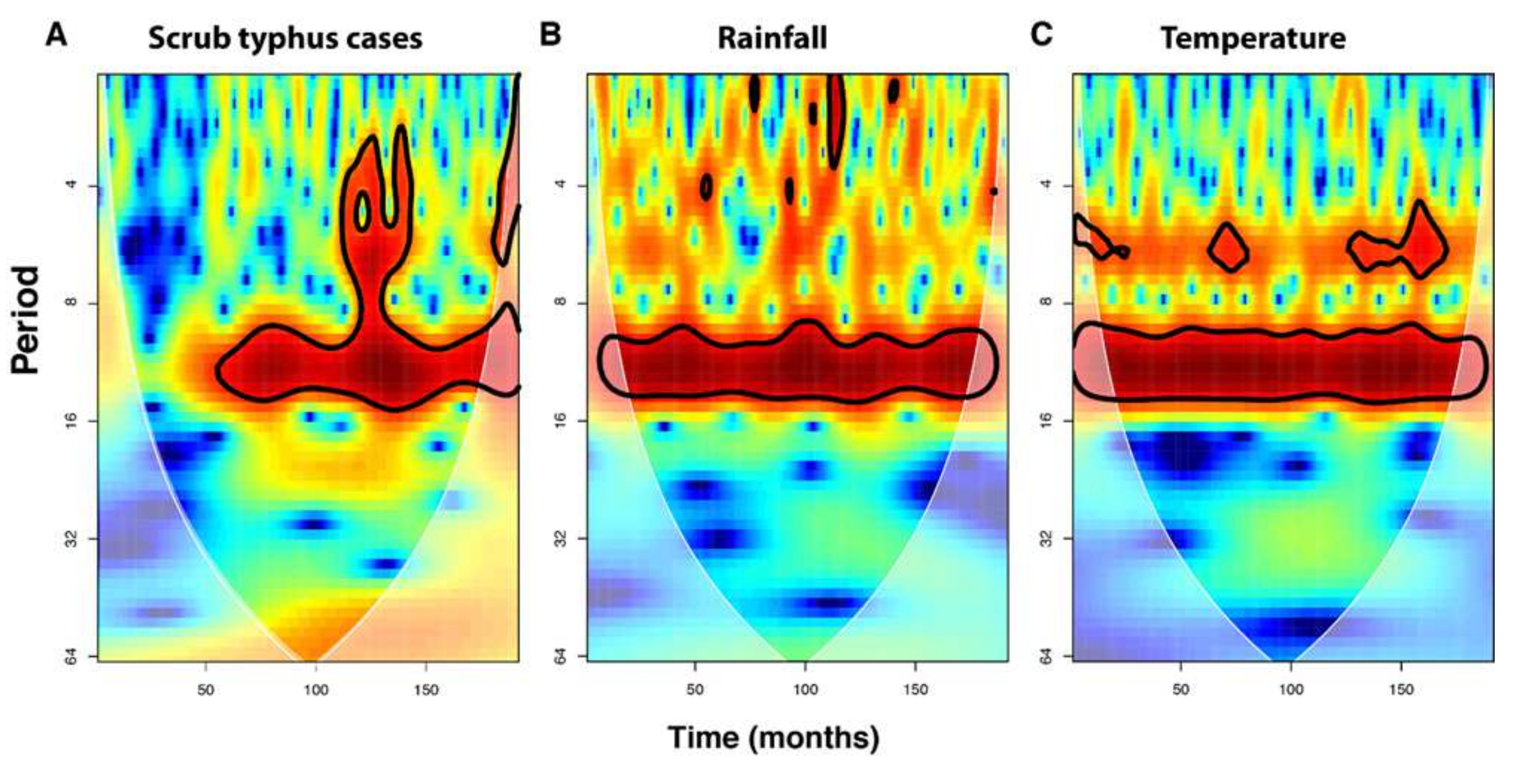

Supplement: S4 Fig — Wavelet analysis for Chiangrai province depicting (A) seasonal pattern of scrub typhus cases from 2008 onwards, (B) significant 12-month periodicity of total monthly rainfall from 2003–2018 and (C) significant 12-month periodicity of average monthly temperature from 2003–2018. (TIF) [file pntd.0008233.s007.tif]

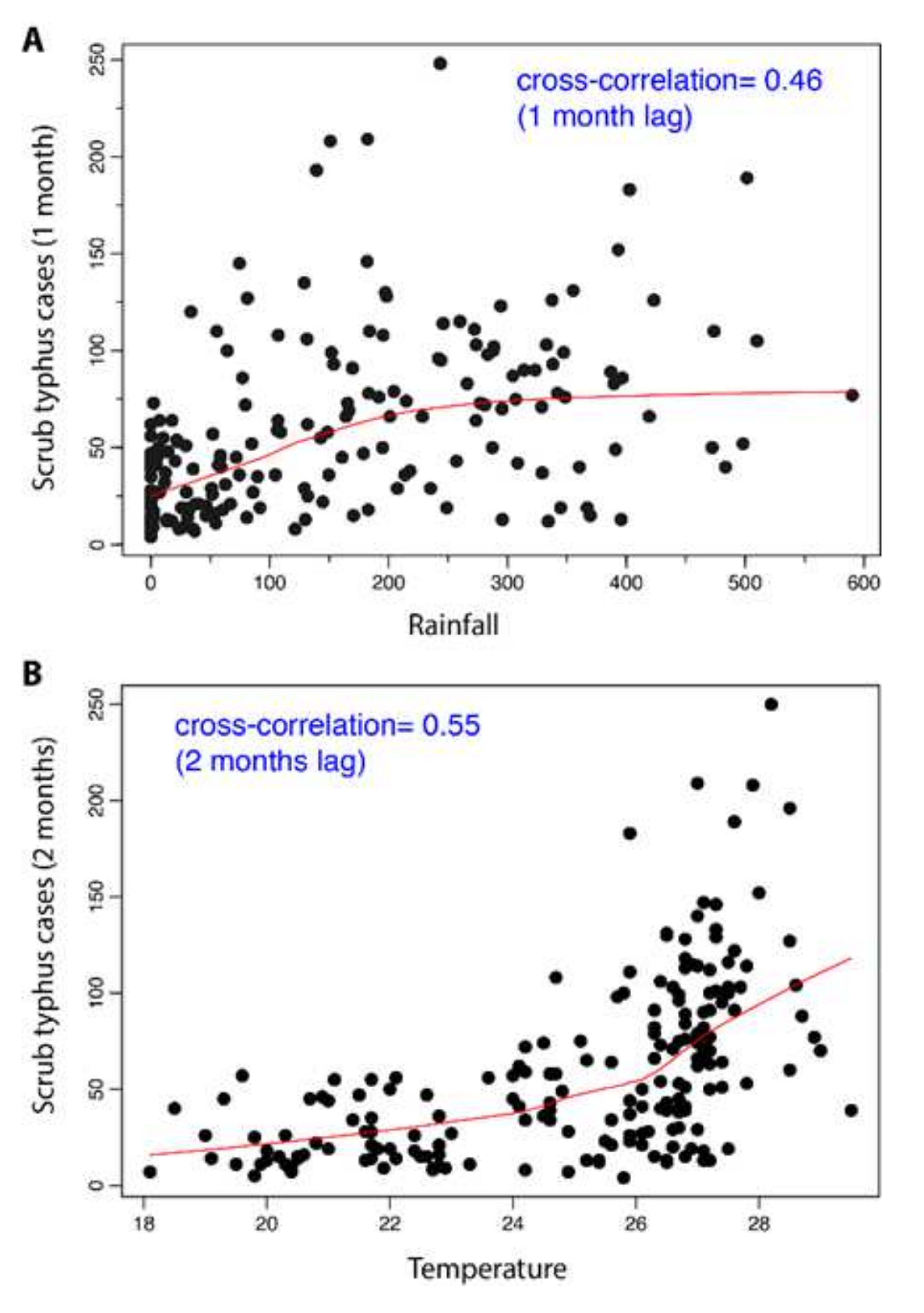

Supplement: S5 Fig — Significant correlation was seen between rainfall and scrub typhus cases (R = 0.46, A) with a lag time of one month along with temperature and scrub typhus cases (R = 0.55, B) with a lag time of two months. (TIF) [file pntd.0008233.s008.tif]

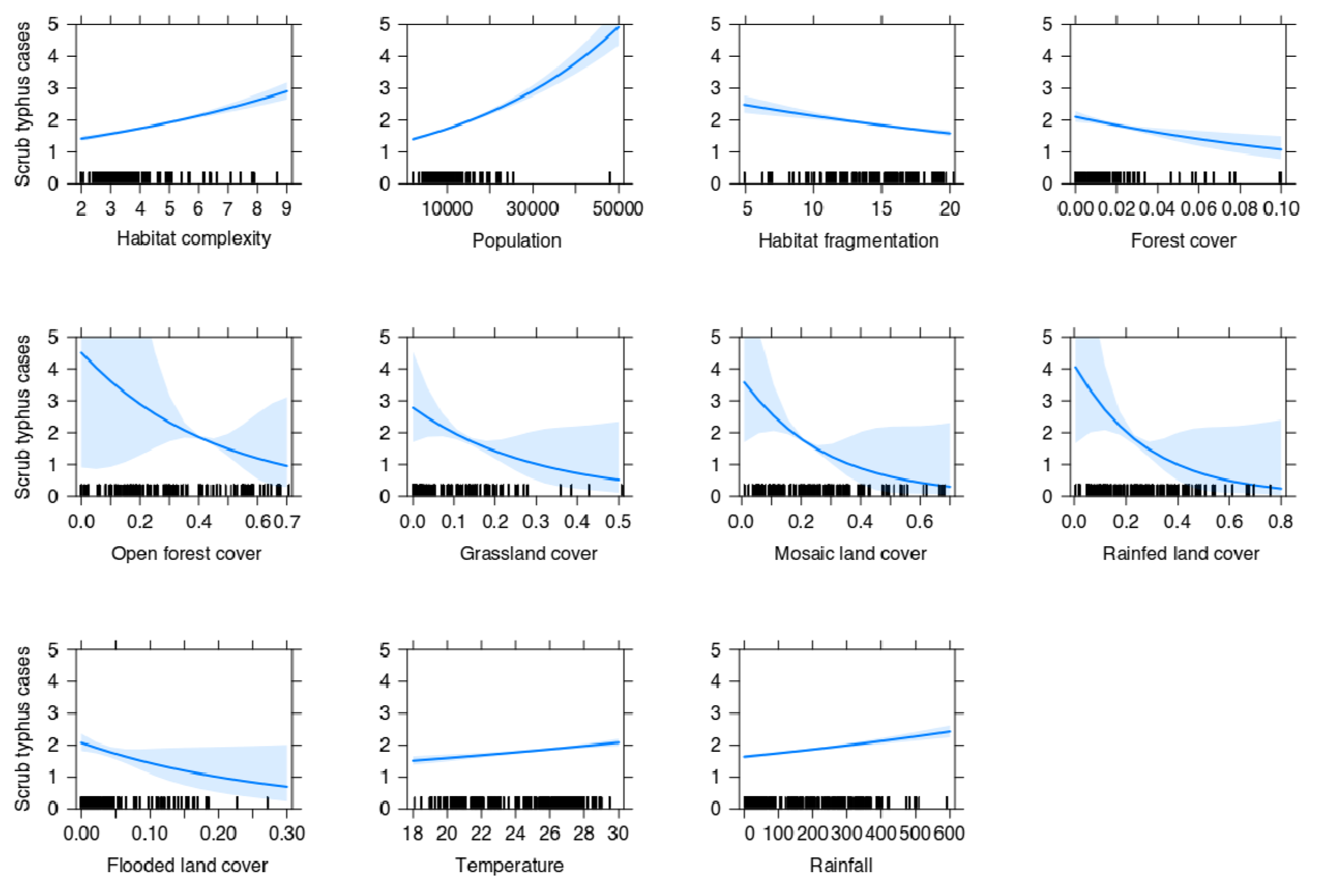

Supplement: S6 Fig — The smoothed variables selected in the best GLM were (A) habitat complexity, (B) population, (C) habitat fragmentation, (D) forest cover, (E) forest open cover, (F) grassland open cover, (G) mosaic habitat cover, (H) rain-fed land cover, (I) flooded-irrigated land cover, (J) average monthly temperature in°C and (K) total monthly rainfall in mm. (TIF) [file pntd.0008233.s009.tif]
